# P-525. HIV/STI Screening and Prevention in Hospitalized Patients with Opioid Use Disorder

**DOI:** 10.1093/ofid/ofae631.724

**Published:** 2025-01-29

**Authors:** Manasa Brown, En-Ling Wu, Mim Ari, George Weyer, Anna Czapar

**Affiliations:** University of Chicago, Chicago, Illinois; University of Chicago, Chicago, Illinois; University of Chicago, Chicago, Illinois; University of Chicago, Chicago, Illinois; University of Chicago, Chicago, Illinois

## Abstract

**Background:**

Patients with opioid use disorder (OUD) are at increased risk of acquiring HIV and HCV. While this association is well-established for injection drug use (IDU), HCV and HIV transmission risk may be elevated among people with OUD in the absence of IDU. In this project, we reviewed rates of HIV, HCV, syphilis, gonorrhea, and chlamydia screening, as well as pre-exposure prophylaxis (PrEP) prescription for patients with OUD to assess baseline screening and prescribing practices and target areas for improvement.

**Figure 1.**
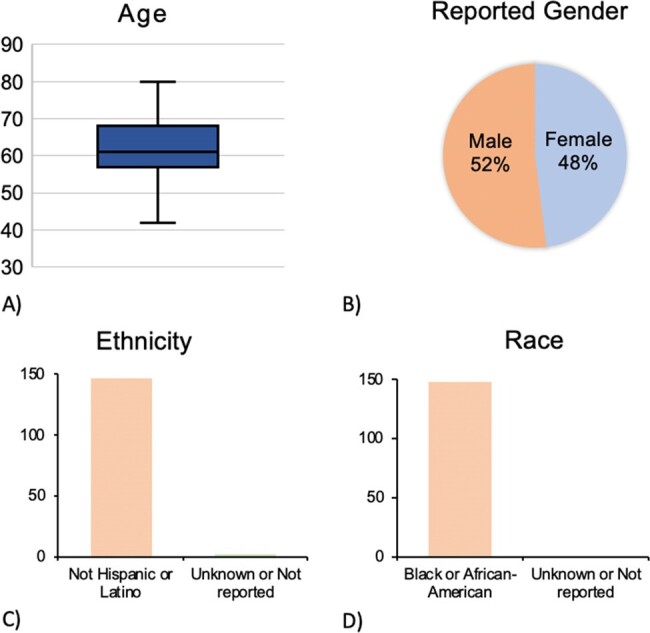
Demographics of Patients Reviewed Demographic summary of patients with OUD reviewed. A) Age at time of admission. B) Patient reported gender. C) Ethnicity of patients evaluated. D) Patient reported race of patients evaluated.

**Methods:**

This project was formally determined to be quality improvement, not human subjects research, and was not overseen by an Institutional Review Board, per institutional policy. We used chart review of a convenience sample of 150 hospitalized patients over the prior 6 months at University of Chicago with diagnosed OUD to characterize patient demographics, substance (or opioid) use patterns, testing for HIV, HCV, gonorrhea, chlamydia, and syphilis in the last year, and PrEP prescriptions or conversations regarding PrEP.

**Figure 2.**
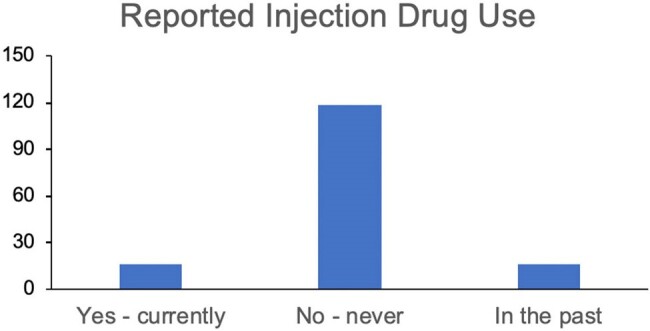
Reported Injection Drug Use Among Patients Evaluated Reported injection drug use was determined by chart review. Patients were characterized as reporting current injection use, prior injection use, or never having used injection opioids.

**Results:**

The majority of patients were non-Hispanic Black with an equal distribution between men and women (self-reported) and an average age of 62 years (Fig 1). While 118 patients (79%) did not report current IDU, 16 (11%) reported current and 16 (11%) prior IDU (Fig 2). Despite emergency department-based universal screening for HIV and syphilis, 48 (32%) and 71 (48%) of all patients and 6 (19%) and 10 (31%) of patients reporting current or prior IDU were not tested for HIV or syphilis, respectively (Fig 3, 4). It was unclear if screening was not offered or declined. Of 102 patients who received HIV screening, only one patient tested positive and was known to be living with HIV. None of the remaining 149 patients were prescribed or had documented discussion regarding PrEP.

**Figure 3.**
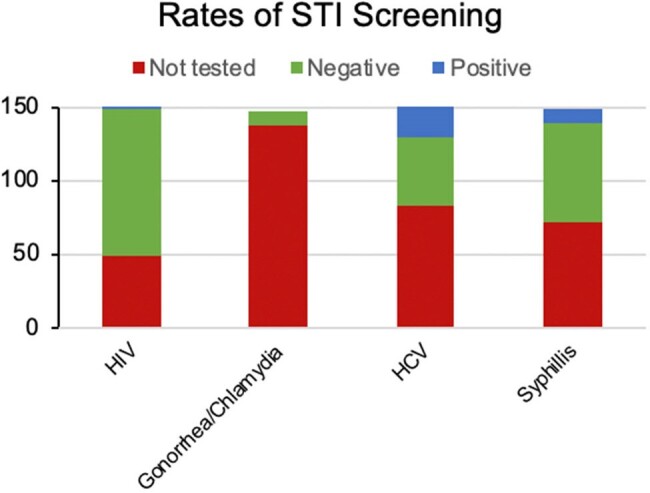
Rates of STI Screening Rates of STI screening for HIV, gonorrhea/chlamydia, hepatitis C, and syphilis were reviewed for all patients with confirmed OUD.

**Conclusion:**

Even among higher risk groups (IDU, positive syphilis screen), none of the sampled hospitalized patients with OUD were prescribed or had documented discussion regarding PrEP Hospitalization of patients with OUD presents an opportunity to screen for and expand conversations surrounding HIV/STI prevention; varied interventions (e.g. educational, clinical decision support systems) for HIV/STI prevention should be developed and studied.

**Figure 4.**
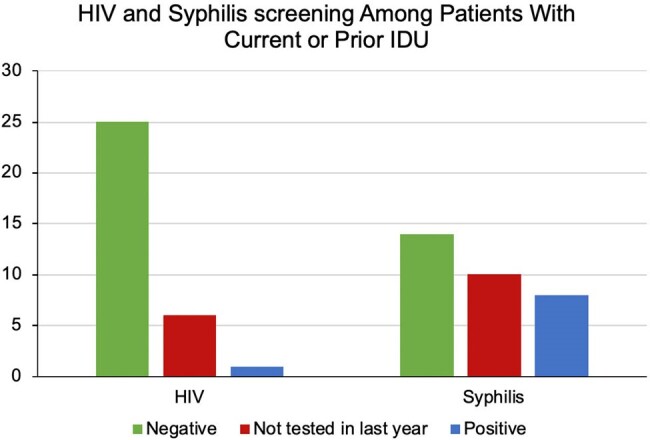
HIV and Syphilis Screening Among Patients with Current or Prior Injection Drug Use Screening rates for HIV and syphilis were evaluated only for patients with a history of current or prior injection drug use.

**Disclosures:**

**All Authors**: No reported disclosures

